# Intracavernous injection in the treatment of erectile dysfunction after radical prostatectomy: an observational study

**DOI:** 10.1590/S1516-31802001000400004

**Published:** 2001-07-07

**Authors:** Joaquim de Almeida Claro, José Elêrton de Aboim, Marcelo Maríngolo, Enrico Andrade, Wilson Aguiar, Marcos Nogueira, Archimedes Nardozza, Miguel Srougi

**Keywords:** Prostate cancer, Erectile dysfunction, Self-injection therapy, Radical prostatectomy, Câncer da próstata, Disfunção erétil, Auto-injeção intracavernosa, Prostatectomia radical

## Abstract

**CONTEXT:**

Despitethe recent improvements in performing radical retropubic prostatectomy that have led to a considerable decrease in the complication rate, erectile dysfunction still represents a major problem. Moreover, less invasive treatment options that are emerging for erectile dysfunction have not shown satisfactory results in managing these patients.

**OBJECTIVE:**

To study the efficacy and side effects of self-injection therapy in the treatment of men who had become impotent after undergoing radical prostatectomy due to prostate cancer, over a study period of 96 months.

**DESIGN:**

Observational study.

**SETTING:**

University Referral Center.

**PARTICIPANTS:**

168 patients with erectile dysfunction, aged 43 to 78 years old, who underwent radical retropubic prostatectomy due to localized prostate cancer.

**PROCEDURES:**

The patients were treated with self-injection therapy using papaverine, phentolamine and prostaglandin E1, at home.

**RESULTS:**

This study showed an acceptable 94.6% success rate, with no life-threatening complications.

In addition to this, our series presented a 13.1% cure rate with this therapy.

**CONCLUSION:**

Self-injection therapy with papaverine, phentolamine and prostaglandin E1 is effective and safe in the treatment of erectile dysfunction after radical prostatectomy.

## RESUMO

**CONTEXTO:**

Apesar do recente avanço técnico na realização da prostatectomia radical retropúbica, que levou a queda no índice de complicações, a disfunção erétil ainda é um problema importante. Além disso, as novas opções (menos invasivas) de tratamento da disfunção erétil não mostram resultados satisfatórios nesse grupo de pacientes.

**OBJETIVO:**

Avaliar a eficácia e efeitos colaterais da auto-injeção no tratamento da disfunção erétil causada pela prostatectomia radical, durante 96 meses.

**TIPO DE ESTUDO:**

Estudo observacional.

**LOCAL:**

Universidade Federal de São Paulo/ Escola Paulista de Medicina.

**PARTICIPANTES:**

168 pacientes com disfunção erétil, entre 43 e 78 anos, submetidos a prostatectomia radical retropúbica, devido a câncer localizado da próstata.

**PROCEDIMENTOS:**

Os pacientes foram tratados com auto-injeção utilizando a associação de papaverina, fentolamina e prostaglandina E1 em casa.

**RESULTADOS:**

O índice de sucesso foi de 94,6% com complicações bastante discretas. Além disso, tivemos um índice de cura de 13,1% nesse estudo.

**CONCLUSÕES:**

A auto-injeção com papaverina, fentolamina e prostaglandina E1 é eficaz e segura no tratamento da disfunção erétil causada pela prostatectomia radical.

## INTRODUCTION

Despite the recent improvements in performing radical retropubic prostatectomy that have led to a considerable decrease in its complication rate, erectile dysfunction still represents a major problem.^1^ Even with properly trained professionals, erectile dysfunction rates range dramatically according to the evaluation of the patients. Although the complete loss of erection had become less frequent after the emergence of the nerve-sparing technique,^2^ some authors have reported up to 75% mild to moderate erectile dysfunction due to this surgery.^3^

In addition to the marked advance in surgical technique, the treatment of erectile dysfunction has also changed profoundly over the last two years, due to the advent of oral drugs, especially sildenafil.^4^

Unfortunately, experiences using this modern and less invasive method in the treatment of impotence caused by radical prostatectomy have been disappointing,^5^ as have been the poor results obtained using MUSE (transurethral alprostadil) for the same purpose.^6^

Considering our previously published^7^^,^^8^ excellent results from using self-injection therapy in the treatment of impotence in patients with unknown etiology, our aim was to evaluate the response to self-injection therapy in those patients submitted to radical prostatectomy due to localized prostate cancer.

## METHODS

A retrospective clinical study was conducted on 168 men who were impotent because of radical retropubic prostatectomy. This surgery had been performed by many different surgeons in order to treat localized prostate cancer, during a period of 96 months (from May 1, 1991 to May 31, 1999), in a university referral center. To be included in the study, the patients needed to have had normal erectile function prior to surgery, as well as being in a stable relationship. The age of these patients ranged from 43 to 78 years (median of 61 years).

The period between the surgery and the onset of treatment ranged from 4 to 84 months (median of 26 months). The patients had undergone the nerve-sparing technique, with the objective of preserving erectile function.^2^ In 92 patients, at least one neurovascular bundle was preserved and in 61 patients the preservation was bilateral. For 15 patients there was no data available regarding this matter.

Of these 168 patients, 42 had prior treatment using oral sildenafil, 17 received oral yohimbine, 11 used the vacuum device and another 8 patients used transurethral alprostadil therapy (MUSE). None of the patients managed with medicinal treatment had successful outcomes, while among the patients treated with the vacuum device there was dissatisfaction with the method.

The only new procedure that the 168 patients underwent was the intracavernous injection of 0.2 ml of a solution of papaverine (22.6 mg), phentolamine (1.34 mg) and prostaglandin E1 (13.4 mg) in the consultation office, as described previously.^9^

After this, the patients were trained to perform the self-injection at home and to return every 30 days to the office. An emergency telephone number was given to the patients to be used in case of side effects or priapism.

## RESULTS

All the 168 patients presented full erections during the office test. The duration of these erections ranged from 20 to 210 minutes (median of 30 minutes). Based upon these results, the initial dose to be used at home was chosen. The mean dose for each patient ranged from 0.2 ml to 0.4 ml, with a minimum dose of 0.1 ml and maximum dose of 0.8 ml.

At home, the success rate (sexual intercourse with a hard erection) was 94.6%. All patients reported mild pain or discomfort and about 15% developed penile ecchymosis during the first three or four self-injections at home, which is believed to have been due to the patients’ learning period. Only 19 patients (11.3%) reported prolonged erection, lasting from 3 to 10 hours. Among these 19 patients only 3 reported erections lasting over 6 hours, although not full. In these cases, conservative management was conducted. All episodes of prolonged erections were resolved spontaneously, at the patients’ homes. The prolonged erection episodes were all reported in the first three months of self-injection therapy.

During the treatment, 22 patients (13.1%) recovered full erections without any therapy. The ages of these 22 patients ranged from 43 to 57 years (median 55 years) after 5 to 18 months (median of 14 months) of treatment and 7 to 22 months (median of 9 months) after radical prostatectomy.

The follow-up ranged from 5 to 72 months (median 29 months). Except among patients for whom therapy was unsuccessful, the self-injection therapy was performed by all patients during the whole follow-up period, with at least one injection each week. Nevertheless, there was wide variation in injection frequency during the study, although the patients were warned to avoid injections on two consecutive days. Neither penile fibrosis nor nodes were observed during the follow-up.

## DISCUSSION

Sexual dysfunction is a well-recognized complication of abdominal and pelvic surgery. As most of these procedures are performed on elderly patients, there has in the past been a tendency to disregard the importance of loss of potency. However, some men now have expectations of unimpaired sexual function well into their seventh or even eighth decades. Impotence can, therefore, have a profound effect on their quality of life.

On the other hand, as prostatic carcinoma continues to undergo a real increase in incidence^1^ and diagnosis tends to be made at earlier stages than in the past,^10^ an increasing number of men are being offered treatment by radical prostatectomy, usually via the retropubic route. Traditionally, impotence was inevitable after this procedure. However, in the early 1980's it was demonstrated that erectile dysfunction was caused by injury to the branches of the pelvic plexus that innervate the corpora cavernosa.^11^ The so-called nerve-sparing technique allows the identification and preservation of the bilateral neurovascular bundle, when compatible with disease control.^2^

Younger patients tend to fare better in terms of postoperative potency than their older counterparts.^12^

Most patients recover potency after radical prostatectomy within 6 to 12 months,^3^ although improvement can continue for up to 2 years postoperatively.

On the other hand, self-injection therapy with the association of papaverine, phentolamine and prostaglandin E1 is successful in up to 100% of the general impotent male population.^13^

So, our success rate of 94.6% was quite predictable. In the same way, painful erections occur in 20.6% of patients during testing and only in 2.9% after this period.^14^ However, our rate of prolonged erections (11.3%) is much higher than the published data of 3.2%,^14^ probably due to the dosage used by our patients.

Interestingly, it has been suggested that early intracavernous therapy, starting one month after surgery, results in an improved potency rate after radical prostatectomy.^15^ This and the latency time of up to 2 years for potency recovery may easily explain our cure rate of 13.1% with self-injection therapy.

However, non-controlled bias could have occurred in the results, as this was an observational study. The patients’ psychological profile with respect to their commitment to treatment could be questioned, as well as their educational level, leading to better results in both cases.

## CONCLUSIONS

The modern oral therapy and transurethral system for erectile dysfunction used by 67 patients (39.9%) in our series, prior to self-injection therapy, did not present a satisfactory success rate.

On the other hand, self-injection therapy using papaverine, phentolamine and prostaglandin E1 represents a good option for the treatment of men with erectile dysfunction after radical prostatectomy. The success rate is quite high (94.6%) including patients with a cure rate of about 13%, with very mild side-effects that resolve spontaneously.
